# Predicting compatibilized polymer blend toughness

**DOI:** 10.1126/sciadv.adk6165

**Published:** 2024-06-19

**Authors:** Robert J. S. Ivancic, Debra J. Audus

**Affiliations:** Materials Science and Engineering Division, National Institute of Standards and Technology, Gaithersburg, MD 20899, USA.

## Abstract

Polymer blends can yield superior materials by merging the unique properties of their components. However, these mixtures often phase separate, leading to brittleness. While compatibilizers can toughen these blends, their vast design space makes optimization difficult. Here, we develop a model to predict the toughness of compatibilized glassy polymer mixtures. This theory reveals that compatibilizers increase blend toughness by creating molecular bridges that stitch the interface together. We validate this theory by directly comparing its predictions to extensive molecular dynamics simulations in which we vary polymer incompatibility, chain stiffness, compatibilizer areal density, and blockiness of copolymer compatibilizers. We then parameterize the model using self-consistent field theory and confirm its ability to make predictions for practical applications through comparison with simulations and experiments. These results suggest that the theory can optimize compatibilizer design for industrial glassy polymer blends in silico while providing microscopic insight, allowing for the development of next-generation mixtures.

## Teaser

A physics-based model predicts compatibilized glassy polymer blends’ toughness, accelerating compatibilizer optimization.

## INTRODUCTION

Polymer blends are ubiquitous in modern technology as they often yield materials with improved properties for a fraction of the cost of designing previously unidentified monomers or polymerization routes (*1*). These materials are used in everyday consumer goods such as packaging, automobiles, and electronics insulation (*2*). They also have applications in many fields, such as tissue scaffolding (*3*), biodegradable packaging (*4*), solar cells (*5*), medical textiles (*6*), and gas separation (*7*). Despite these materials’ success, their design space is limited due to their typically brittle behavior. This lack of toughness directly results from their low entropy of mixing, which scales as the inverse of the molecular mass. This low barrier results in blends that phase separate on the micrometer scale. A lack of molecular bridges spanning phase-separated components causes them to fracture easily.

For example, polypropylene and polyethylene, two common commodity polymers, fracture at 300 and 800% strain, while their blend fractures at only 10% strain (*8**,* *9*). Similarly, mixing glassy polymers, such as polystyrene (PS) and poly(methyl methacrylate) (PMMA), leads to a decrease in fracture toughness by more than a factor of 20 (*10*). Improving the toughness of immiscible polymers would benefit society greatly across various domains. For instance, high-quality blends could decrease plastic recycling costs by up to 95% by reducing the need for precision sorting (*11*). This application would promote sustainability and provide a crucial avenue for solving the world’s plastic pollution crisis (*12*).

Adding a small amount of compatibilizer is one way to improve the toughness of polymer blends. They accomplish this by stitching the phase-separated domains together themselves or broadening the interface, allowing more interactions between homopolymers. Compatibilizer architectures vary greatly. Janus particles (*13*), grafted nanoparticles (*14*), and graft copolymers (*9**,* *15*) increase the interface’s fracture energy. Diblock (*16*–*18*), random (*19**,* *20*), or multiblock (*8**,* *10**,* *21*) copolymers are also commonly used. In these cases, sequence substantially affects the toughness of compatibilized blends. Given this broad design space, how to choose a compatibilizer to meet a target toughness is unclear.

Simulation and theory have provided several valuable insights; however, considerable challenges remain. While many simulation studies evaluate these interfaces’ thermodynamics (*22*–*24*) or dynamics (*25*–*27*), few studies consider how compatibilizer architecture affects a blend’s long-timescale, post-yield mechanical properties, such as toughness. In one such case, Brown (*28*) created a solid mechanics model that predicts glassy polymer blend toughness scales with the square of the areal density of diblock compatibilizers. This model, designed for the crazing regime in which the compatibilizers can deform the homopolymers into a fibril network, suggests that this scaling is due to cross-tie fibrils. Although this prediction holds for diblocks that are several entanglement lengths long, it requires modifications for shorter blocks (*16*). How to apply these predictions to other compatibilizer architectures remains an open question.

Our work builds a theory that predicts the toughness of any glassy compatibilized blend using basic information about the polymers, such as the Flory-Huggins parameter (χ) and entanglement length (*N_e_*) along with the chosen compatibilizer architecture and concentration. We can break down this model into two parts. First, a mechanics model reveals the physics of compatibilization on a microscopic level. Second, we parameterize this model using self-consistent field theory (SCFT) simulations that allow for efficient use of this model. Unlike Brown’s model, we create this model at the single chain level based on well-validated bulk toughness models for single-component glassy polymers. Furthermore, we limit ourselves to the crazing rather than the chain scission regime (*29*) in which compatibilizers sever across the interface without deforming the polymer matrix. We take this approach because crazing is substantially more effective at imbuing toughness to a blend as it requires both chain scission and homopolymer deformation, making it more industrially relevant.

We use both extensive molecular dynamics and extant experiments to validate this model. Our theory allows for the quick iteration of any compatibilizer design in silico to meet a target toughness for glassy and rubbery polymer blends. While crystallization will likely complicate the picture in semicrystalline blends, our model may also serve as a qualitative framework. As such, our theory provides valuable insights for designing compatibilizers for modern technology that requires tough polymer blends.

## RESULTS

### Mechanics model

**Fig. 1.**
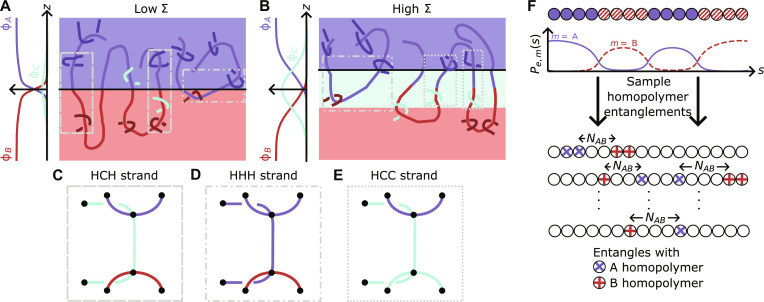
Model schematics. (**A** and **B**) Our system under two scenarios: low and high compatibilizer areal density (Σ). The plots on the left show the volume fractions of homopolymer A (blue; ϕ*_A_*), homopolymer B (red; ϕ*_B_*), and compatibilizer C (green; ϕ*_C_*) as a function of the distance from the interface (*z*). In the diagrams on the right, thick lines in medium hue represent a given polymer, while the dark red, blue, and green kinks represent load-bearing entanglements with A, B, and C. In (A), the fracture plane (black line) is at the AB interface. In (B), enough compatibilizer has built up at the AB interface to create a C domain between the A and B phases, moving the fracture plane to the AC interface. (**C** to **E**) Types of load-bearing strands, where black dots represent entanglements between polymers and blue, red, and green colored lines show A, B, and C polymer segments between these entanglements. They carry stress across the fracture planes. Example HCH, HHH, and HCC load-bearing strands are boxed in (A) and (B) with dashed, dash-dotted, and dotted lines. (**F**) A tetrablock compatibilizer made of A (blue monomers) and B (red striped monomers) with the probability of monomer *s* entangling with a polymer of type *m*, *P*_*e*,*m*_(*s*) below it. *P*_*e*,*m*_(*s*) is calculated from Eq. 7 using SCFT. These probabilities are used to simulate load-bearing entanglements with many compatibilizers. From these sampled compatibilizers, we directly measure the mean length (〈*N_AB_*〉) and frequency of load-bearing strands from the A to B domain per compatibilizer. Applying Eq. 2 and using Σ allows for the direct computation of $M_{\mathrm{HCH}}{\langle \tilde{N}\rangle }_{\mathrm{HCH}}$ . Simulated entanglements with compatibilizers, which are necessary to determine if an entanglement is load-bearing, are not shown for clarity.

In this section, we will show how to predict the toughness of a compatibilized polymer blend from a set of microscopic structural variables before deformation. We consider a glassy, incompatible blend of homopolymers A and B along with a compatibilizer C, as shown in Fig. 1 (A and B). For simplicity, we assume the bonds along all polymer backbones are independent of chemistry, and the mixture is far enough below the glass transition temperature so that disentanglement is negligible. We strain this system perpendicular to the interface and focus on the crazing regime where inelastic deformation occurs before chain breaking. In this scenario, a fracture plane will initiate, and polymer segments that span the fracture plane and form strong entanglements on both sides of this plane will support stress across it. We illustrate these segments in Fig. 1 (C to E) and denote them as “load-bearing strands.” We label these load-bearing strands with three letters. The outer letters represent whether the entanglements form with compatibilizers (C) or homopolymers (H), and the interior letter denotes whether the connecting strand is a C or H.

Inspired by prior works (*30*–*33*), our model suggests that the energy to break all the load-bearing strands that bear stress on the interface is

$$\Gamma_{\text{model}}=\underset{f}{\text{min}}\left(\epsilon \sum_{t\in f}‍M_{t}{\langle \tilde{N}\rangle }_{t}-\Gamma_{0}\right)$$ (1)

Here, *f* represents a potential fracture plane, ϵ is proportional to the statistical energy to break a bond, *t* is the type of strand as illustrated in Fig. 1 (C to E), *M_t_* is the areal density of load-bearing strands of type *t*, ${\langle \tilde{N}\rangle }_{t}$ is the mean length of load-bearing strands of type *t*, and Γ_0_ is a small positive energy barrier. The tilde over the *N* differentiates this length, which includes contributions from three different polymers, from lengths along a single polymer, denoted by the variable *N*. We emphasize that the first term inside the min function of Eq. 1 is in analogy to prior theories for fracture by Mikos and Peppas (*30*) for homopolymer glasses and by Lake and Thomas (*31*) for polymer networks, where one treats cross-linkers as load-bearing entanglements. These ideas have been used in subsequent theories, such as Wool’s percolation model of polymer fracture (*32*), and verified numerous times (*33**,* *34*). While previous theories consider homogeneous materials and only have a single type of load-bearing strand, the complexities of our three-component system require that we consider multiple types. The summation ( $\sum_{t\in f}‍M_{t}{\langle \tilde{N}\rangle }_{t}$ ) represents the areal density of the length of all load-bearing strands crossing the fracture plane and is based on the same physical principles as prior single-component theories.

The first term in the min of Eq. 1 suggests that the fracture energy is proportional to three quantities. First, it is proportional to the number of load-bearing strands (*M_t_*) such that more load-bearing strands increase the fracture energy. Second, it is proportional to the length of the load-bearing strands ( ${\langle \tilde{N}\rangle }_{t}$ ) because all bonds in the strand must be stretched to their breaking points before a single bond breaks. Last, Eq. 1 multiplies this product by the statistical energy from breaking a bond (ϵ). This statistical energy does not directly correspond to the energy to break a single bond. First, theoretically, ϵ is proportional to the energy stored in a bond of a load-bearing strand, which is different than the energy to break a bond due to the fact that the strand is under tension. This phenomenon is known as the “assisted jumper” mechanism (*35*). Second, not all the bonds may stretch the same amount. Third, ϵ includes contributions from the energy to deform the homopolymer inelastically, i.e., to craze it. While more work is needed to obtain ϵ a priori, we simply consider ϵ as the characteristic energy related to breaking a bond in a polymer strand.

While we expect toughness to scale as $\sum_{t\in f}‍M_{t}{\langle \tilde{N}\rangle }_{t}$ , a solid must overcome a stress barrier to deform beyond its local yield strain, allowing it to craze. Potential fracture planes that cannot overcome this barrier, therefore, must fail via chain scission or pullout. These failure modes require substantially less energy than crazing due to their lack of inelastic deformation of the homopolymer matrix. To account for this phenomenon, the second term inside the min of Eq. 1 is a small energy density (stress) barrier (Γ_0_ > 0). In the chain scission or pullout regimes below this threshold ( $\sum_{t\in f}‍M_{t}{\langle \tilde{N}\rangle }_{t}<\Gamma_{0}/\epsilon$ ), we expect that our model does not hold. A prior work has assumed a similar barrier to crazing (*16*). Later, we show that Γ_0_ is independent of compatibilizer architecture, entanglement length, and interfacial width. These observations suggest that this constant is due to the local glassy structure of the material rather than its polymeric nature.

In Eq. 1, we must identify the fracture plane (*f*) with the minimum fracture energy since we assume this is where rupture occurs. Three potential fracture plane candidates exist in our system. The most apparent potential fracture plane is the AB interface (*f* = AB), as shown in Fig. 1A. In this case, the main contribution of load-bearing strands will be the compatibilizer stitching the A homopolymer phase to the B homopolymer phase. As the compatibilizer areal density (Σ) increases, the compatibilizer will start segregating, and the fracture energy will increase due to two effects. First, the number of load-bearing strands at the interface (*M_t_*) will increase. Second, these strands will become longer ( ${\langle \tilde{N}\rangle }_{t}$ ) to pass through segregated compatibilizers at the interface and anchor into the homopolymer domains. As the AB fracture plane becomes stronger, two additional potential fracture planes emerge: the interface between the A homopolymer and compatibilizer, the AC interface (*f* = AC), as shown in Fig. 1B, and the interface between the B homopolymer and the compatibilizer, BC interface (*f* = BC), which is the mirror image of the AC case. These fracture planes occur because Σ is large enough that compatibilizer segregates from the homopolymer matrices. While load-bearing strands are often more numerous on the AC and BC interfaces (larger *M_t_*) compared to the AB interface, they are typically shorter (smaller ${\langle \tilde{N}\rangle }_{t}$ ) as they only need to span from the homopolymer matrix to the nearby compatibilizer domain.

Before accounting for the length and number of load-bearing strands of these three planes, we briefly clarify the meaning of the “strong” entanglements that are necessary to create a load-bearing strand. Following Bukowski *et al.* (*33*), we define a strong, “load-bearing entanglement” as any entanglement that is not a part of either associated polymer’s first or last primitive paths. Thus, they exclude the initial and final entanglements of the kinking polymer. In addition, they do not involve entangling around any monomer present in the first or last primitive paths of the other polymer. Examples of such strands are shown in Fig. 1 (C to E).

We now consider the AB fracture plane and enumerate load-bearing strands of type *t* ∈ *AB* in Fig. 1A. All load-bearing strands that connect the A to the B homopolymer matrices must break when this interface fractures. This requirement yields two types of load-bearing strands. The first type, HCH, occurs when a compatibilizer spans load-bearing entanglements with homopolymers A and B (see Fig. 1C). Because this case corresponds to the low Σ regime in which a compatibilizer domain has not stabilized and thus cannot bear stress, we ignore any compatibilizer-compatibilizer entanglements, as shown in the rightmost example of an HCH load-bearing strand in Fig. 1A. The second type, HHH, occurs when a homopolymer spans load-bearing entanglements with homopolymers A and B (see Fig. 1D). Treating each of the five polymer segments that make up an HCH load-bearing strand (see Fig. 1C) as a spring allows us to apply parallel and series spring rules (see the Materials and Methods section for details) to calculate the load-bearing strand length as

$${\langle \tilde{N}\rangle }_{\mathrm{HCH}}=\langle N_{\mathrm{AB}}\rangle +\frac{1}{2}\left(N_{e,A}+N_{e,B}\right)$$ (2)

Similarly,

$${\langle \tilde{N}\rangle }_{\mathrm{HHH}}=\frac{1}{2}\left[\text{max}\left(N_{e,A},N_{e,B}\right)+3\;\text{min}\left(N_{e,A},N_{e,B}\right)\right]$$ (3)

Here, 〈*N_AB_*〉 is the mean length between A and B entanglements in our compatibilizer, and *N*_*e*,*A*_ (*N*_*e*,*B*_) is the length between entanglements in homopolymer A (homopolymer B). In general, these lengths should be measured in units such that it requires the same force to break one unit of A as a unit of B so that they maintain the same meaning. However, because we assume all polymers have the same backbone bonds, we simply measure these lengths in monomers throughout this piece.

We now must determine how often each type of load-bearing strand occurs to compute the fracture energy for the AB interface. The HHH load-bearing strands occur whenever a homopolymer entangles with a homopolymer of the opposite type. Hence, the number of these strands is proportional to *Z_AB_*/2, where *Z_AB_* is the areal density of entanglements between species A and B. The factor of 1/2 occurs to avoid double-counting entanglements between these species. Because the number of HCH strands depends substantially on the architecture of the compatibilizer, we denote the areal density of these strands as *M_HCH_*. Thus

$$\sum_{t\in \text{AB}}‍M_{t}{\langle \tilde{N}\rangle }_{t}=M_{\mathrm{HCH}}{\langle \tilde{N}\rangle }_{\mathrm{HCH}}+\frac{1}{2}Z_{\mathrm{AB}}{\langle \tilde{N}\rangle }_{\mathrm{HHH}}$$ (4)

Because the AB interface may not have the lowest fracture energy, we also consider the AC interface in Fig. 1B. In this scenario, we assume that the compatibilizer begins to segregate from both homopolymer phases, i.e., high Σ. When the AC interface fractures, the load-bearing strands must connect the A homopolymer phase to the C compatibilizer phase or the homopolymer B phase beyond the C compatibilizer phase. This results in two varieties of load-bearing strands. The first type is HCC, in which a compatibilizer strand forms load-bearing entanglements with homopolymer A and compatibilizer C (see Fig. 1E). These load-bearing strands are relevant, unlike the low Σ case, because enough compatibilizer has segregated at the interface to stabilize it, allowing the domain to bear stress. The second type, HHH, as described above, occurs when a homopolymer strand crosses the compatibilizer C phase to form a load-bearing entanglement with the opposite homopolymer phase (see Fig. 1D). In principle, a third variety of load-bearing strands, HCH strands, exists as in the AB fracture interface case. However, these strands are unlikely to occur since the compatibilizer strand has a high probability of entangling with another compatibilizer before entangling with a B homopolymer due to the substantial concentration of compatibilizer at the AB interface, as shown in the right example of HCC strands in Fig. 1B. Thus, these compatibilizer strands are already accounted for as HCC strands. Again, using spring rules, the Materials and Methods section shows

$${\langle \tilde{N}\rangle }_{\mathrm{HCC}}=2N_{e,A}$$ (5)

We now must determine how often each type of load-bearing strand occurs to compute the fracture energy. The number of HHH load-bearing strands is the same as before. The HCC load-bearing strands occur whenever a homopolymer entangles with a compatibilizer. Hence, these strands are proportional to *Z_AC_*/2, where *Z_AC_* is the areal density of entanglements between species A and C. Again, the factor of 1/2 is to avoid double-counting. Thus, the total fracture energy for the AC interface is

$$\sum_{t\in \text{AC}}‍M_{t}{\langle \tilde{N}\rangle }_{t}=\frac{1}{2}Z_{\mathrm{AC}}{\langle \tilde{N}\rangle }_{\mathrm{HCC}}+\frac{1}{2}Z_{\mathrm{AB}}{\langle \tilde{N}\rangle }_{\mathrm{HHH}}$$ (6)

An analogous expression holds for the BC interface.

Now, toughness, a dynamic variable, can be predicted by the microscopic, structural variables of a system prior to its deformation via Eqs. 1 to 6.

### Validation of the mechanics model

Molecular dynamics simulations provide a direct way to validate this model, albeit at high strain rates. To this end, we performed extensive molecular dynamics simulations of glassy, immiscible polymer blends compatibilized with multiblock copolymers. Our simulations use a coarse-grained polymer model in which bonds can break. For simplicity, we keep the two homopolymers symmetric with the same intraspecies interactions, same entanglement lengths (*N_e_*), and same chain lengths (*N*); the compatibilizer chain length is also *N*. To fully test the theory, several parameters are varied. The interspecies interactions, parameterized by the Flory-Huggins parameter (χ), vary between 0.11 and 2.54 as measured by fitting interfacial width profiles of the uncompatibilized blend and assuming a reference volume of the size of the monomer, as described in the Supplementary Materials. We show the fits in fig. S6. We vary chain stiffness via bending rigidity such that *N_e_*, as measured by the Z1 code (*36*), ranges between 10.3 and 37.0 monomers. This range of Flory-Huggins parameters and chain stiffnesses encompasses most commodity polymer blends (*37*). We also increase *N* with *N_e_* to maintain approximately the same number of entanglements per chain. Details about simulations with constant *N* but varying *N_e_* can be found in fig. S5. We consider compatibilizers with *n_b_* = 2 to 32 blocks where each block has equal length *L* = *N*/*n_b_*. We vary compatibilizer areal density $\Sigma =0.0075/\sigma_{\mathrm{LJ}}^{2}$ to $\Sigma =0.045/\sigma_{\mathrm{LJ}}^{2}$ where σ*_LJ_* is the monomer diameter, often taken as ~1 nm. Additional molecular dynamics simulation details are in the Materials and Methods section.

**Fig. 2.**
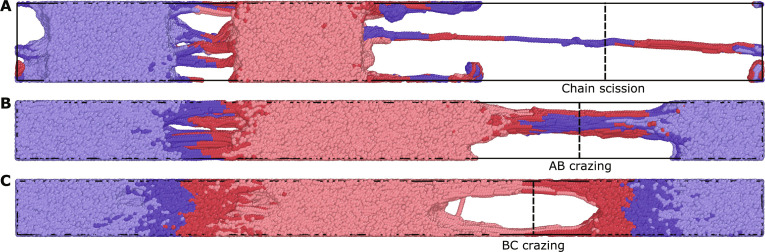
Molecular dynamics simulations with different fracture modes. Polymer blend compatibilized with a hexablock compatibilizer at 300% strain at increasing different areal densities of compatibilizer, (**A**) $\Sigma =0.0075/\sigma_{\mathrm{LJ}}^{2}$ , (**B**) $\Sigma =0.015/\sigma_{\mathrm{LJ}}^{2}$ , and (**C**) $\Sigma =0.045/\sigma_{\mathrm{LJ}}^{2}$ . Blue and red show polymers of types A and B, respectively. Compatibilizers have a darker hue to distinguish them from homopolymers. These images show three different fracture mechanisms: chain scission, AB crazing, and BC crazing, respectively. Snapshots were generated using OVITO (*61*).

This large design space (χ, *N_e_*, *N*, *n_b_*, and Σ) allows for rigorous testing of the theory. Furthermore, it allows us to investigate a broad set of failure modes. Figure 2 shows molecular dynamics simulation snapshots at 300% strain. The lowest Σ, Fig. 2A, shows only a small amount of homopolymer deformation before failure, suggesting a transitional state between the chain scission and crazing regimes. As Σ increases, Fig. 2B shows that the AB interface has fractured. Unlike Fig. 2A, homopolymer deformation is more robust, indicating that this failure mode shows AB crazing. At the highest Σ in Fig. 2C, crazing still occurs. Instead of the AB interface failing, the interface between homopolymer B and the compatibilizer fails, demonstrating that BC crazing occurs at this compatibilizer concentration. These images provide qualitative evidence of the multiple failure modes suggested in the proposed mechanics model.

To test the mechanics model quantitatively, we explicitly determine entanglements in our molecular dynamics simulations using the Z1 code (*36**,* *38*–*40*) that reduces our polymer configurations to their primitive paths by straightening them without allowing them to cross. At the end of this algorithm, we define entanglements as the set of “kinks” where the chains bend around neighboring chains. We deem the chains that entangle with these entanglements as the nearest neighbor chains to these kinks. More details about this procedure can be found in the Materials and Methods section. This process allows us to calculate explicitly all microscopic variables used in our mechanics model.

**Fig. 3.**
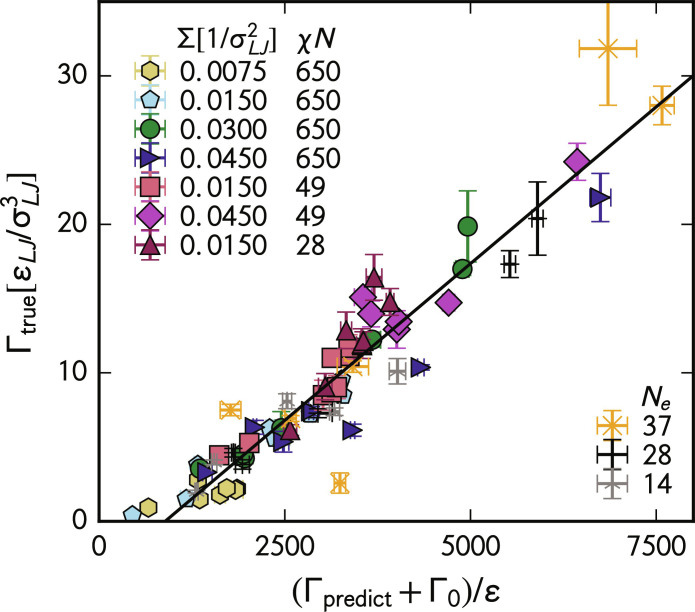
Explicit test of the mechanics model. The true toughness against the scaled predicted toughness of our molecular dynamics simulations. The upper left shows a legend for data points in which we varied compatibilizer areal density (Σ) and Flory-Huggins parameter (χ*N*) but fixed the entanglement length to *N_e_* = 10.3 and chain length to *N* = 256 repeat units. The bottom right shows a legend for data points in which we varied the number of entanglements per chain *N_e_* but fixed compatibilizer areal density to $\Sigma =0.015/\sigma_{\mathrm{LJ}}^{2}$ and the interspecies interactions to be the same as the χ*N* = 650 case. In this case, we keep the number of entanglements per chain roughly equal by increasing *N*. Data points with the same symbol represent blends compatibilized with block copolymers with different block lengths *L*. We plot these architectures against their toughness as symbols in Fig. 4. Error bars are SEs of three replicates.

We plot the scaled predicted toughness [(Γ_model_ + Γ_0_)/ϵ] versus true toughness (Γ_true_) for our dataset in Fig. 3. The predicted toughness comes from Eq. 1. We calculate the true toughness of these blends by integrating the stress-strain curve of our simulations until failure, defined as when the stress in the direction of pull goes to 0. Specifically, we integrate the stress tensor in the direction of stretching, σ, from a strain of 0 until the strain at which σ = 0, ϵ_failure_, i.e., $\Gamma_{\text{true}}=\int_{0}^{\epsilon_{\text{failure}}}‍\sigma d\epsilon$ . Each point in the figure represents an average of three replicate molecular dynamics simulations for a given compatibilized blend. Error bars represent SEs of the predicted and true toughnesses. The error in predicted toughness comes from variance in the microscopic variables in the simulations, while the error in true toughness comes from this variance and thermal fluctuations. The legend markers in the upper left show data in which we fix entanglement length while we vary the areal density and Flory-Huggins parameter. The legend markers in the lower right show data in which we specify compatibilizer areal density and interspecies interaction parameter while we vary entanglement length.

Overall, our model compares excellently with the microscopic data. The predictions correlate strongly with the actual values, with a Pearson correlation coefficient of *r* = 0.95. The line is the best fit. The error bars demonstrate that most architectures we test have replicates on either side of the line. These predictions show the wide range of validity of our microscopic mechanics model. Again, we emphasize that our parameters cover many industrially relevant cases, suggesting that this model should apply to experimental data. While a few points reside more than 3 SEs away from the line, these errors do not appear systematic. It remains unclear whether these points are simply outliers due to our study’s limited statistics or whether a higher-order theory could capture the toughness of these simulations.

As predicted by the model, the *x* intercept of Fig. 3 is positive, demonstrating an energy density barrier to crazing. This barrier is constant, even with changing compatibilizer architecture, *N_e_*, and χ. This effect is similar to the assumption of a stress barrier proposed by Creton *et al.* (*16*). As this barrier does not depend on the polymeric or interfacial properties, we expect that it is related to the microscopic yield strains seen in theories of glass mechanics (*41**,* *42*).

### SCFT parameterization

As derived, the mechanics model provides a microscopic picture of how compatibilizers toughen an interface. However, up to this point, the model relies on expensive molecular dynamics simulations to parameterize five microscopic quantities ( ${\langle \tilde{N}\rangle }_{\mathrm{HCH}}$ , *M_HCH_*, *Z_AB_*, *Z_AC_*, and *Z_BC_*) in addition to knowledge of two material properties (ϵ and Γ_0_). This limitation makes the model difficult to use in practice. Here, we show how to parameterize the model with SCFT simulations. The benefit of using SCFT to determine these microscopic variables is its speed. We can perform these simulations in seconds using a modern central processing unit (CPU). Compared to molecular dynamics, SCFT simulations run a factor of 10^5^ faster for comparable systems. The drawback is that this approach requires several additional assumptions about the nature of entanglements at the interface.

We make four simplifying assumptions to complete this task tractably. First, we assume that each layer a distance *z* from the interface is well mixed. This statement guarantees that sufficient time has passed such that the compatibilizers have equilibrated at the interface and that fluctuations are not large enough to invalidate the SCFT’s mean-field assumption. This assumption is reasonable in the regime of interest but will break down as we approach the order-disorder transition in which fluctuations strengthen or when equilibration time is insufficient. Second, we assume that the number of entanglements between two polymer types is proportional to the number of contacts between them. Physically, this assumption means that an entanglement between two polymer types occurs more often when they co-occur at high concentrations. Third, we assume that the entanglement length does not change as a function of *z*. This notion ensures that the rate of entanglements on a given polymer type is constant, regardless of the distance from the interface. Last, we assume that these entanglements’ positions are independent of position along the chains, i.e., entanglements are no more or less likely to occur near other entanglements. Later, we will show that these assumptions are approximately valid in many industrially relevant cases.

We first seek to determine where load-bearing entanglements occur in our compatibilizer chains. Under the above assumptions, the probability for a monomer *s* along the backbone of a compatibilizer to form a load-bearing entanglement with a polymer of type *m* a distance *z* away from the interface is ϕ*_m_*(*z*)/*N*_*e*,*C*_(*s*), where *m* can be homopolymer A (*m* = *A*), homopolymer B (*m* = *B*), or compatibilizer C (*m* = *C*). Here, *N*_*e*,*C*_(*s*) = {*N*_*e*,*A*_ if the type of *s* is *A*, *N*_*e*,*B*_ otherwise} is the local entanglement length. The function ϕ*_m_*(*z*) is the volume fraction of load-bearing monomers of *m* at *z*. This function differs from the total volume fraction of *m* at *z* as described in the Materials and Methods section because the chain ends do not contribute.

Using the above expression and rules of conditional probabilities, the probability of forming a load-bearing entanglement at position *s* in the compatibilizer is

$$P_{e,m}\left(s\right)=\frac{1}{N_{e,C}\left(s\right)}\int \mathrm{dz}\;p_{C}\left(z|s\right)\phi_{m}\left(z\right)$$ (7)

where *p_C_*(*z* ∣ *s*) is the conditional probability of monomer *s* being distance *z* from the interface. All functions in the integrand are directly calculable using the propagators, which are key functions in SCFT. Further details are provided in Materials and Methods.

Using the expression for *P*_*e*,*m*_(*s*), we simulate entanglements between many compatibilizers and other polymer species, as shown in Fig. 1F. We then directly measure the mean number ( ${M\prime }_{\mathrm{HCH}}$ ) of HCH load-bearing strands per compatibilizer and 〈*N_AB_*〉, which allows for the computation of the mean length of HCH load-bearing strands ( ${\langle \tilde{N}\rangle }_{\mathrm{HCH}}$ ) via Eq. 2. By multiplying ${M\prime }_{\mathrm{HCH}}$ by the areal density of compatibilizers at the interface, we obtain $M_{\mathrm{HCH}}={M\prime }_{\mathrm{HCH}}\Sigma$ . These quantities allow us to evaluate the first term of Eq. 4.

Having determined the first two microscopic quantities, we must parameterize *Z_AB_*, *Z_AC_*, and *Z_BC_*. Using the same assumptions as above, we find

$$Z_{\mathrm{AB}}=\left(\frac{\rho_{A}}{N_{e,A}}+\frac{\rho_{B}}{N_{e,B}}\right)\int \mathrm{dz}\;\phi_{A}\left(z\right)\phi_{B}\left(z\right)$$ (8)

$$Z_{\mathrm{AC}}=\left(\frac{2\rho_{A}}{N_{e,A}}\right)\int \mathrm{dz}\;\phi_{A}\left(z\right)\phi_{C}\left(z\right)$$ (9)

hold with an analogous formula for *Z_BC_*. Here, ρ*_m_* is the number density of species *m*. These quantities allow for the evaluation of the other terms in Eqs. 4 and 6.

Given these expressions, we explicitly test the last two assumptions, which could not be entirely rationalized a priori. As demonstrated in fig. S1, we show that, while the independence assumption holds, the entanglement length does vary as a function of the distance from the interface in the first few Kuhn lengths, leading to quantitative errors in applying this model. Recent molecular dynamics simulations near block copolymer interfaces see similar changes in the number of entanglements on approach to the interface (*43**,* *44*). Brown and Russell (*45*) suggest that this variance is due to confinement changing the pervaded volume of the polymers. These errors are generally small compared to the variations in toughness in many industrially relevant regimes.

### Validation of the SCFT parameterization

We now validate the SCFT parameterization of the microscopic variables in our mechanics model. This section uses the finite differences variant of the open-source polymer self-consistent field software package (*46**,* *47*) to run our field-based simulations and obtain the propagators.

**Fig. 4.**
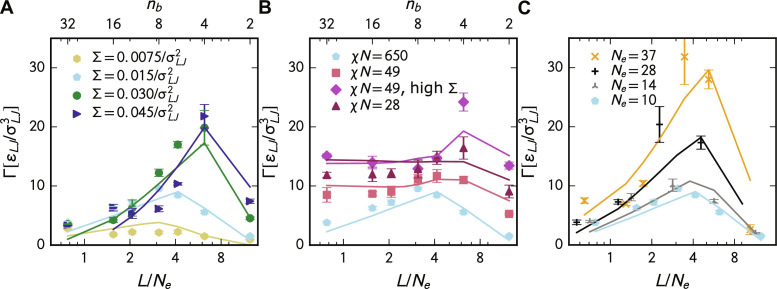
SCFT parameterization of mechanics model. The toughness (Γ) against block length measured in monomers normalized by entanglement length (*L*/*N_e_*) for changing (**A**) compatibilizer areal density, (**B**) Flory-Huggins parameter, and (**C**) entanglement length. The markers correspond to the symbols in Fig. 3 and show the molecular dynamics simulations’ true toughness (Γ_true_). Error bars show the SE of three replicates. Lines represent SCFT parameterized mechanics model predictions. The number of blocks (*n_b_*) is denoted on the upper axis of subplots (A) and (B).

In Fig. 4, we first test our SCFT parameterization by plotting true toughness (Γ_true_) against block length normalized by the entanglement length (*L*/*N_e_*). The error bars in these plots are the SE of three replicate molecular dynamics simulations. The lines connect SCFT parameterized mechanics model predictions taken at the same *L*/*N_e_* as the molecular dynamics simulations. Here, ϵ and Γ_0_ are fit once to all the data. The SCFT parameterized model aligns with the data, capturing toughness trends with *L*, Σ, χ, and *N_e_*. Under the 10 conditions studied, the optimal number of blocks was predicted five times by the SCFT parameterized model. An additional four times, the SCFT parameterized model predicted a sequence within two blocks (*n_b_* = *N*/*L*). Thus, the model is powerful for rapid parameter sweeps to optimize compatibilization.

Despite the overall success of the model, the deviations from the data in these plots should be addressed. The areal density of HCH load-bearing strands (*M_HCH_*) is slightly overpredicted using this method, leading to an overprediction of AB crazing energy. The technique also slightly undercounts the areal density of homopolymer-compatibilizer entanglements (*Z_AC_* and *Z_BC_*). This leads to an underestimation of homopolymer-compatibilizer fracture energy. More details about these errors can be found in fig. S2. These two factors, which are due to deviations from our assumptions, lead to systematic errors in Fig. 4, which are substantially larger than the errors in Fig. 3. These errors show the limits of using SCFT to parameterize our microscopic variables, not the limits of our mechanics model.

Moving beyond simulated data, several experimental observations suggest the validity of this model. Eastwood and Dadmun (*10*) studied the fracture energy interfaces of PMMA and PS compatibilized with multiblock copolymers. They find that this energy degrades substantially when the block length of their compatibilizer is less than 2*N_e_*, approximately the same scale seen here. Similarly, Brown (*28*) suggests that Γ ∝ Σ^2^ for long diblocks. Experiments that measured the fracture energy of poly(phenylene oxide) and PMMA interfaces motivated this theory. In this work, the interfaces are compatibilized with varying amounts and molecular weights of diblock PMMA-PS. Our molecular dynamics simulations and model capture a similar scaling relationship, as shown in fig. S3.

**Fig. 5.**
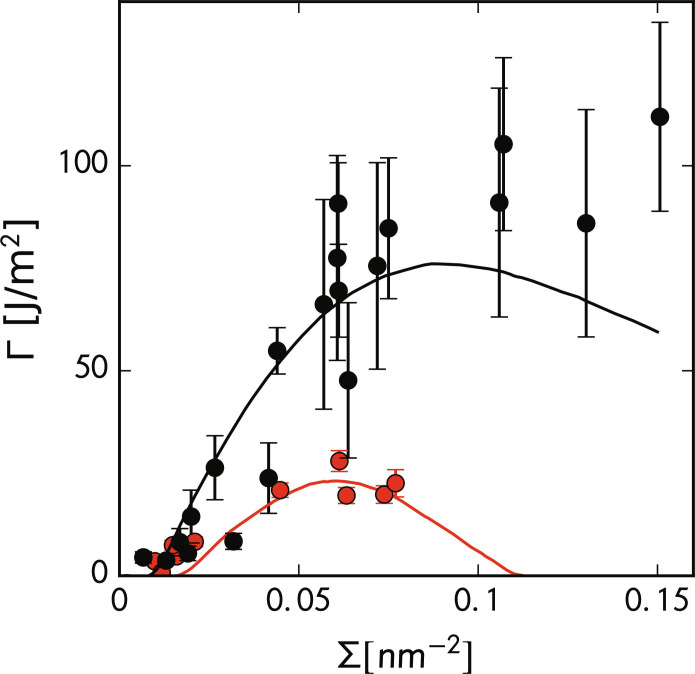
Experimental test of SCFT parameterized mechanics model. The fracture energy against the areal density of compatibilizers (Σ) for two different diblock architectures of PS-P2VP diblocks: 500 to 540 repeat units (red) and 800 to 870 repeat units (black). Because of statistical sampling, we apply a Savitzky-Golay filter with a window size of 0.014 nm^−2^ and a polynomial order of 3 to smooth a small amount of noise in our SCFT parameterized model lines. Error bars in the experimental data points represent 1 SD of roughly 20 measurements. Experimental data were taken from Creton *et al.* (*16*).

More explicitly, we fit our model to experimental data of Creton *et al.* (*16*). They performed experiments in which PS and poly(2-vinylpyridine) (P2VP) were compatibilized with diblocks of varying sizes. Because P2VP is chemically identical to PS except for the substitution of a single heavy atom nitrogen for a carbon, we will assume that these species have the same stiffness ( $\langle R^{2}\rangle /M=0.437\;{Å}^{2}\;\text{mol}/g$ ) and entanglement length (*N_e_* = 128 repeat units) (*37*). Noting that the Flory-Huggins parameter of this mixture is about 0.11 assuming a reference volume of $200\;{Å}^{3}$ (*16**,* *48*) and fitting the ϵ and Γ_0_ parameters gives the result in Fig. 5.

The fits show that the model quantitatively predicts the fracture energy of the experiments with only two chemistry-dependent fitting parameters, ϵ and Γ_0_. Specifically, the position and height of the plateau in toughness are predicted fairly accurately. At high areal compatibilizer density (Σ), our model predicts a downturn in the fracture energy. This is due to the reduced number of load-bearing strands as the compatibilizer domain more fully separates from the homopolymer domains. We do not fully understand the experimental phenomenon, but one potential explanation is increased surface roughness at high Σ. As shown by Zhang *et al.* (*49*), the high areal density of compatibilizer can lead to substantial interfacial roughening. This effect could effectively lengthen the interface, leading to a decrease in local Σ. Although we expect our mechanics model to hold, such fluctuations are not captured in SCFT, leading to a breakdown in our predictions at high Σ.

As a final point, computing this model was quite computationally efficient. Calculating the high-resolution lines pictured required approximately 2 hours total or equivalently ~30 s per point on a single CPU. For comparison, a single molecular simulation, which is equivalent to a point, takes around 450 hours of CPU time in the prior section.

### Trends in the simulation data

Here, we briefly assess the various trends observed using molecular dynamics simulations, as seen in Fig. 4. First, as block length (*L*) increases, i.e., the number of blocks (*n_b_* = *N*/*L*) decreases, toughness first increases and then drops. This trend occurs because we maintain a constant compatibilizer length (*N*). At high *L*, blocks can easily anchor into the homopolymer matrix, but only a few blocks exist. This effect limits the number of load-bearing strands between the A and B phases. For example, a diblock contributes essentially one load-bearing strand in the high segregation limit. Decreased *L* increases the number of blocks at constant *N*, toughening the blend. This effect initially increases the number of load-bearing strands; however, eventually, these strands become too short to anchor into the homopolymer phases. This effect decreases blend toughness at small *L*.

Figure 4A also generally shows that toughness increases as the compatibilizer areal density (Σ) grows. The mechanics model suggests that this effect occurs for two reasons. First, raising Σ boosts the number of load-bearing strands that cross the interface. Second, the compatibilizer begins to shield itself from homopolymer entanglements. This shielding yields longer load-bearing strands in compatibilizers with long enough *L*. Thus, the compatibilizer *L* at which the blend is the toughest increases with growing Σ.

Toughness increases with decreasing Flory-Huggins parameter (χ) in Fig. 4B. Decreasing the Flory-Huggins parameter causes homopolymers to become more miscible, raising the number of HHH load-bearing strands and toughening the blend. Last, increasing the entanglement length (*N_e_*) increases toughness by lengthening load-bearing strands when the number of entanglements per compatibilizer is constant, as shown in Fig. 4C. When *N*, rather than *N*/*N_e_* is kept constant, toughness can increase or decrease with increasing *N_e_* depending on the block length *L* as shown in fig. S5. Another key takeaway is that the optimal number of blocks in the compatibilizer, while often not a diblock, depends on the material system defined by χ and *N_e_*.

## DISCUSSION

In this work, we have designed a model that uses experimental data such as entanglement length (*N_e_*) and Flory-Huggins parameter (χ) to predict the toughness of a compatibilized glassy polymer blend quickly. We can divide this model into two parts. The first part is a mechanics model similar to models proposed by Lake and Thomas (*31*) and Mikos and Peppas (*30*). This theory is validated using extensive molecular dynamics simulations in Fig. 3. The second uses additional assumptions to parameterize this mechanics model via SCFT. We test this combined theory using our molecular dynamics simulations in Fig. 4 and extant experimental compatibilized blend data in Fig. 5. In both cases, comparisons are in good agreement with the theory demonstrating the power of our method.

Our model provides a framework for tuning the toughness of a compatibilized polymer blend. For glassy and rubbery mixtures, we can apply these results directly to technologies such as toughening high-impact PS (*50*), increasing its durability, or membranes for gas purification (*7*), increasing their lifetime. We have made the code to run the SCFT parameterized model publicly available to facilitate this application. While we have shown that this theory works for block copolymer compatibilizers, SCFT simulations and the concept of load-bearing strands can be trivially extended to other types of compatibilizers, e.g., random copolymer, graft copolymer, or grafted nanoparticle compatibilizers. For semicrystalline blends, several additional complications, such as the effect of the crystal on deformation energies and the potential for homopolymer-compatibilizer cocrystallization, mean that our model does not directly apply. Despite these issues, our model may provide a conceptual framework for this problem as the notion of load-bearing strands, regardless of whether stabilized via entanglements or crystallization, appears quite general in analogy to tie chains. Interfacial toughening should occur when the number or length of these strands increases.

Our model and molecular dynamics simulations also shed light on the microscopic mechanisms of glassy polymer fracture. Although our mechanics model is quite similar to the theory proposed by Mikos and Peppas (*30*), subtle differences warrant further discussion. The concept of load-bearing entanglements introduced by Bukowski *et al.* (*33*) is vital to obtaining correct toughness predictions at small compatibilizer lengths. We explicitly test this concept by performing simulations of short diblocks in fig. S4. Our results support that entanglements with the first and last primitive paths bear little mechanical load. Another difference, the energy density barrier to form a craze (Γ_0_), distinguishes our mechanics model from prior work (*30*, *31*). This parameter is identifiable as the molecular dynamics data’s *x* intercept in Fig. 3. Mikos and Peppas (*30*), which one can consider a scaling theory, contains no such barrier. The microscopic structural origins of this parameter remain mysterious. However, we suspect that the local packing of the glass may play a substantial role.

As demonstrated, this theory allows for the rapid testing of various compatibilizers in silico. Here, we propose several potential research directions to improve our method. Our SCFT parameterized model is semiquantitative over a large parameter space, but exact predictions remain elusive. These errors occur because our model’s parameterization assumptions break down. To address this issue, one could consider how entanglement length varies near a compatibilized interface, which we find to cause the most prominent errors in our parameterization.

Furthermore, we may relax the well-mixed assumption by using more advanced field theoretical methods that account for the fluctuations in the volume fractions so long as the system is equilibrated. In experiments, we expect that this equilibration assumption is only sometimes applicable, especially when large amounts of compatibilizer are in the blend. In these cases, dynamics play an essential role in the effectiveness of a given compatibilizer in toughening the mix, and therefore, interfacial adhesion energy is a strong function of equilibration time (*51*). Inferior compatibilizers that can more readily localize to the interface may better toughen mixtures in finite time than superior, unequilibrated compatibilizers. Thus, the diffusion and self-assembly of compatibilizers near the interface is a critical research area.

## MATERIALS AND METHODS

### Length of load-bearing strands

We first work to derive the expression for Eq. 2, the length of the HCH load-bearing strand. Consider the vertical load-bearing strand in Fig. 1C. The vertical, compatibilizer segment comprises *N_AB_* bonds. The number of monomers between the A-C (B-C) entanglement and each of the A (B) entanglements is *N*_*e*,*A*_ (*N*_*e*,*B*_). These A and B segments stabilize the load-bearing entanglements with the compatibilizer. We now assume that the bonds are harmonic and that the energy for a bond to break is $\epsilon =k\Delta x_{b}^{2}/2$ , where *k* is a spring constant and Δ*x_b_* is the extension. When the load-bearing strand is stretched, the same force acts on all vertical compatibilizer bonds, while half of this force acts on the A and B stabilizing segments, leading the bonds in these segments to displace half as much. Using spring rules, one obtains

$$\begin{array}{cc} E_{\mathrm{HCH},\text{break}} & =N_{\mathrm{AB}}\left(\frac{k}{2}\Delta x_{b}^{2}\right)+2N_{e,A}\left[\frac{k}{2}{\left(\frac{\Delta x_{b}}{2}\right)}^{2}\right]\\ & +2N_{e,B}\left[\frac{k}{2}{\left(\frac{\Delta x_{b}}{2}\right)}^{2}\right]=\left(\frac{2N_{\mathrm{AB}}+N_{e,A}+N_{e,B}}{2}\right)\epsilon \end{array}$$ (10)

Because $E_{\mathrm{HCH},\text{break}}=\epsilon {\langle \tilde{N}\rangle }_{\mathrm{HCH}}$ for a single load-bearing strand, this expression demonstrates Eq. 2. We use analogous methods to derive Eqs. 3 and 5.

### Obtaining *p_C_*(*z* ∣ *s*) and ϕ*_m_*(*z*) from SCFT propagators

Solving the modified diffusion equation in SCFT yields two propagators, $q_{m}\left(z,s\right)$ and $q_{m}^{†}\left(z,s\right)$ for each polymer *m* subject to the initial conditions of $q_{m}\left(z,0\right)=q_{m}^{†}\left(z,N\right)=1$ . Because the product of compatibilizer propagators, $q_{C}\left(z,s\right)q_{C}^{†}\left(z,s\right)$ , is proportional to $p_{C}\left(z|s\right)$ (*46*), applying the appropriate normalization gives

$$p_{C}\left(z|s\right)=\frac{q_{C}\left(z,s\right)q_{C}^{†}\left(z,s\right)}{\int \mathrm{dz}\;q_{C}\left(z,s\right)q_{C}^{†}\left(z,s\right)}$$ (11)

To obtain ϕ*_m_*(*z*), we seek the volume fraction of polymer *m* that are not in the first or last primitive path. A good approximation of this criteria is the set of polymer monomers more than one entanglement length away from a chain end. Under the assumption of incompressibility, we obtain

$$\phi_{m}\left(z\right)=\frac{1}{NQ_{m}}\int_{N_{e,m}}^{N-N_{e,m}}‍\mathrm{ds}\;q_{m}\left(z,s\right)q_{m}^{†}\left(z,s\right)$$ (12)

where *Q_m_* = (1/*L_z_*) ∫ *dz q_m_*(*z*, *N*).

### Molecular dynamics simulation details

We model semiflexible coarse-grained bead-spring polymers using a 12–6 Lennard-Jones potential for nonbonded interactions. We measure mass, energy, and length in monomer mass (*m_LJ_*), well depth (ϵ*_LJ_*), and bead diameter (σ*_LJ_*) units. We truncate this interaction at *R_c_* = 2.5σ*_LJ_* and shift the potential to maintain continuity. We show that this choice has a minimal effect on blend toughness as discussed in fig. S7. Using $\epsilon_{\mathrm{LJ}}^{\mathrm{AB}}=0.5\epsilon_{\mathrm{LJ}}$ , 0.97ϵ*_LJ_*, and 0.985ϵ*_LJ_*, we obtain interfacial profile widths for noncompatibilized blends that we fit using the equation by Semenov (*52*) to get the Flory-Huggins parameters as in fig. S6. We take intrachain bonded interactions via the finitely extensible nonlinear elastic (FENE) potential with standard parameters (*53*). The bond angle potential is a cosine potential that is minimized when the three monomers form a line, i.e., *U_a_*(θ) = κ[1 − cos (θ)], where θ is the angle between bond vectors. The strength of this potential takes the values of κ = 2ϵ*_LJ_*, 1.5ϵ*_LJ_*, 0.75ϵ*_LJ_*, and 0ϵ*_LJ_*. These values correspond to *N_e_* = 10.3, 14.1, 28.1, and 37.0 monomers, respectively. We obtain these values by running the Z1 code on homopolymer simulations at the corresponding stiffnesses. To keep *N*/*N_e_* roughly constant, we use chain lengths *N* = 256, 320, 512, and 768, in each of these cases, respectively.

To equilibrate our polymers, we first carefully design an initial configuration. Our initialization begins in a periodic box with box lengths $L_{z}=2L_{x}=2L_{y}$ and a target density of $\rho =0.82\sigma_{\mathrm{LJ}}^{-3}$ with 102,400 monomers. We seek to equilibrate these polymers at a temperature of *T* = 1ϵ*_LJ_*/*k_B_*. To obtain the initial homopolymer configurations, we use a Monte Carlo approach that fixes bond length to the minimum FENE potential location and accounts for bond angle and nonbonded interactions between the next nearest neighbor monomers to grow each chain from a single monomer such that the end-to-end distances match those found in Auhl *et al.* (*54*). We randomly place each homopolymer in the box so the two species are phase separated in the *z* dimension. We initialize compatibilizers similarly; however, their unique architecture requires additional consideration. After making our compatibilizers with the same technique as the homopolymers, we fix their centers of mass at the interface. Assuming that A and B species form density fields with a sharp interface, we calculate the energy of the compatibilizer configurations. We then modify the compatibilizer configuration bond angles using the same potentials as before or rotate the configuration. We use Monte Carlo criteria to determine if we should keep the trial configuration. We repeat this Monte Carlo procedure 10^6^ times at which point we see our energies plateau, indicating equilibration. We then randomly place these compatibilizers in the *xy* plane at the interface. As a final step in generating our initial configurations, we perform 300 small solid rotations and translations to each homopolymer to minimize density fluctuations in the box. We describe this Monte Carlo procedure in greater detail in the Supplementary Materials.

Inspired by Sliozberg and Andzelm (*55*), we run a dissipative particle dynamics (DPD) simulation for 3400τ, where $\tau =\sqrt{\frac{m_{\mathrm{LJ}}\sigma_{\mathrm{LJ}}^{2}}{\epsilon_{\mathrm{LJ}}}}$ , and the conservative force is a combination of the classical repulsive DPD potential with a cutoff radius taken to be the Lennard-Jones minima with the attractive cosine squared potential proposed by Cooke *et al.* (*56*) with a cutoff radius of $\frac{7}{4}\sigma_{\mathrm{LJ}}$ . In particular, our conservative potential is

$$U_{C}\left(r\right)=U_{C,\text{rep}}\left(r\right)+U_{C,\text{att}}\left(r\right)$$ (13)

where

$$U_{C,\text{rep}}\left(r\right)=\left\{\begin{array}{ll} \frac{A}{2^{5/6}}{\left(1-\frac{r}{2^{1/6}\sigma_{\mathrm{LJ}}}\right)}^{2}, & \text{if}\;r\leq 2^{1/6}\sigma_{\mathrm{LJ}}\\ 0, & \text{if}\;r>2^{1/6}\sigma_{\mathrm{LJ}} \end{array}\right\}$$ (14)

and

$$U_{C,\text{att}}\left(r\right)=\left\{\begin{array}{ll} -\left(\epsilon_{\mathrm{LJ}}^{\mathrm{ij}}\right)\prime , & \text{if}\;r\leq \sigma_{\mathrm{LJ}}\\ -\left(\epsilon_{\mathrm{LJ}}^{\mathrm{ij}}\right)\prime \text{cos}{\left[\frac{2\pi }{3}\left(r/\sigma_{LJ}-1\right)\right]}^{2}, & \text{if}\;\sigma_{\mathrm{LJ}}<r\leq \frac{7}{4}\sigma_{\mathrm{LJ}}\\ 0, & \text{if}\;r>\frac{7}{4}\sigma_{\mathrm{LJ}} \end{array}\right\}$$ (15)

We take the energy constants for the repulsive potential to be *A* = 25ϵ*_LJ_* as in (*55*) and the attractive interaction’s same species well depth to be $\left(\epsilon_{\mathrm{LJ}}^{\mathrm{AA}}\right)\prime =\left(\epsilon_{\mathrm{LJ}}^{\mathrm{BB}}\right)\prime =0.2\epsilon_{\mathrm{LJ}}$ . This smaller well depth prevents monomers from unphysically fusing. The cross-interaction well depth is $\left(\epsilon_{\mathrm{LJ}}^{\mathrm{AB}}\right)\prime =0.1$ , 0.17, and 0.185ϵ*_LJ_* for Lennard-Jones cross-interaction well depths of $\left(\epsilon_{\mathrm{LJ}}^{\mathrm{AB}}\right)=0.5$ , 0.97, and 0.985ϵ*_LJ_*, respectively. During this run, we increase angular stiffness by Δκ = 0.2ϵ*_LJ_* to compensate for the softer nonbonded potential. After completing this soft equilibration, we linearly ramp up the constants to *A* = 125ϵ*_LJ_* and $\left(\epsilon_{\mathrm{LJ}}^{\mathrm{ij}}\right)\prime =\epsilon_{\mathrm{LJ}}^{\mathrm{ij}}$ while decreasing Δκ = 0 over 1600τ similarly to Dietz and Hoy (*57*). At this point, the potential looks similar to a soft Lennard-Jones potential. We quickly raise to *A* = 1000ϵ*_LJ_* to ensure that no monomers overlap. Last, we switch to the Lennard-Jones potential and equilibrate in the isothermal-isobaric (NPT) ensemble at a pressure *P* = 0 for an additional 10^4^τ. To check our equilibration, we compared our homopolymers’ mean squared internal distances to that of Auhl *et al.* (*54*). For the compatibilizers, we checked to ensure that the compatibilizer profiles approximated what SCFT predicts. In both cases, our results compared well to these benchmarks.

We lower the temperature from *T* = 1 to 0.4ϵ*_LJ_*/*k_B_*, above the glass transition, at a constant rate of 5 × 10^−5^ϵ*_LJ_*/(*k_B_*τ). Because little equilibration occurs at this rate below this temperature, we then quickly quench from *T* = 0.4 to 0.1ϵ*_LJ_*/*k_B_*. We deform our simulations in the *z* dimension in the NPT ensemble, where the *x* and *y* dimensions are allowed to vary to maintain pressure. This step occurs at a constant velocity of 0.00634σ*_LJ_*/τ, giving a strain rate of approximately 10^−4^/τ, commensurate with other molecular dynamics simulations (*29**,* *58*–*60*). We verified for a select set of simulations that deforming at a rate one order of magnitude slower did not substantially affect the toughness of the simulations. During these simulations, bond length and angle potentials permanently break if the bond length exceeds *r*_break_ = 1.2σ*_LJ_*, i.e., the energy difference between this extension and the minimum exceeds 14ϵ*_LJ_*. This cutoff provides a maximum force of 40 times higher than the force to break interchain bonds, similar in magnitude to previous coarse-grained simulations (*29**,* *58*). In addition, this cutoff was chosen to allow our mechanical deformations to fracture at a reasonably small strain, allowing our simulations to run efficiently. We compare this bond breaking methodology to the Morse potential in fig. S8 and show that this choice does not affect our results qualitatively. These simulations continue until the stress in the *z* direction is less than 0.

### Z1 code analysis

The Z1 code (*36*) fixes the chain ends of the polymers in the system and shrinks them to their primitive paths. This code outputs a list of kinks’ associated polymer, spatial position, and chain location. To determine which polymer each kink wraps, we wrote an analysis code adding points every 0.025σ*_LJ_* for all the primitive paths. Using these points, we identify the polymers close to each kink. We say that the polymer closest to a kink entangles with it. To avoid nonphysical self-entanglements, we do not consider points less than σ*_LJ_* away from the original kink on the same primitive path in this step. We choose the threshold σ*_LJ_*, the diameter of a monomer, as it is the smallest length scale in our simulations. Determining this information allows us to calculate load-bearing strands explicitly.

### Disclaimer

Certain equipment, instruments, software, or materials are identified in this paper to specify the experimental procedure adequately. Such identification is not intended to imply recommendation or endorsement by the National Institute of Standards and Technology nor is it intended to imply that the materials or equipment identified are necessarily the best available for the purpose.
