# Supplementary material for: Predicting compatibilized polymer blend toughness
Source: Sci Adv. 2024 Jun 19;10(25):eadk6165. doi: 10.1126/sciadv.adk6165 (PMC11186489; doi:10.1126/sciadv.adk6165)
Supplement: Supplementary file 1 — Supplementary Text Figs. S1 to S8 [file sciadv.adk6165_sm.pdf]

Science Advances
AAAS

Supplementary Materials for

Predicting compatibilized polymer blend toughness

Robert J. S. Ivancic and Debra J. Audus

Corresponding author: Robert J. S. Ivancic, robert.ivancic@nist.gov.

Sci. Adv. **10**, eadk6165 (2024)
DOI: 10.1126/sciadv.adk6165

This PDF file includes:

Supplementary Text
Figs. S1 to S8

Predicting compatibilized polymer blend toughness: supplemental materials

This text is the supplemental materials for Ivancic and Audus, *Science Advances* (2024). It contains additional information and analysis to support the conclusions in the manuscript. Data for these claims and code are found at https://doi.org/10.18434/mds2-3057.

1. SELF-CONSISTENT FIELD THEORY (SCFT) PARAMETERIZATION

For the SCFT parameterization in the model to hold, four assumptions must be met. One is system-dependent (assumption 1), and another has solid theoretical underpinnings (assumption 2). The other two assumptions require further justification.

We first check assumption 3, that the entanglement length (N_e) does not change as a function of distance from the interface (z) in Fig. S1A. This plot shows $N_e(z)$ for a selection of compatibilizer block lengths (L) for our simulations with an areal density of compatibilizers $\Sigma = 0.045\sigma_{LJ}^{-2}$ and Flory-Huggins parameter $\chi N = 650$. Here, σ_{LJ} is the length scale of the Lennard-Jones minimum. Interestingly, it shows positive and negative fluctuations away from the bulk value of N_e depending on the L and z. While this image clarifies that assumption 3 does not hold exactly, fluctuations away from N_e are less than 30% in all cases that are not immediately adjacent to the interface and often are short ranged. These deviations are even less at lower Σ. Thus, we believe that $N_e(z) = N_e$ is a reasonable assumption in many circumstances.

We next move onto assumption 4, which is entanglements' positions are independent of their position along the chains. To test this claim, we consider the normalized correlation of entanglements a distance s from a given entanglement on a compatibilizer,

$$c(s) = \frac{\langle (1 - P_{e,C}(s'))\,(e(s'-s) - P_{e,C}(s'-s))\rangle}{\langle (1 - P_{e,C}(s'))^2\rangle}, \tag{S1}$$

where

$$e(s) = \left\{ \begin{array}{ll} 1 & \text{if s entangles with a homopolymer} \\ 0 & \text{otherwise} \end{array} \right\}, \tag{S2}$$

$P_{e,C}(s)$ is the probability of monomer s of a compatibilizer entangling, and the average is taken when an entanglement occurs at s'. This function takes a value of 1 when entanglements are completely correlated and 0 when entanglements are completely decorrelated. As shown for a representative compatibilizer in Fig. S1B, we see no correlations other than self-correlations ($s = 0$) in our data, indicating that assumption 4 holds in all cases.

To understand how much assumption 2 affects the model's predictions, we plot the true and predicted microscopic quantities in Fig. S2. First, we consider how these errors affect the AB crazing energy by considering the total length of HCH load-bearing strands per area, $M_{HCH}\langle\widetilde{N}\rangle_{HCH}$, in Fig. S2A. The SCFT parameterization overpredicts these values by around 20%. As this term consists of two parameters M_{HCH} and $\langle\widetilde{N}\rangle_{HCH}$, we can further ask which of these variables causes this overprediction. By plotting the true and predicted values of M_{HCH}, Fig. S2B demonstrates that M_{HCH} is the source of these issues. Thus, we can conclude that the SCFT parameterization overpredicts AB crazing energy because it predicts too many HCH load-bearing strands. Next, we move to the AC-crazing energy. We show that the SCFT model underpredicts the number of AC entanglements in Fig. S2C by about 10%. Thus, the parameterization underpredicts AC crazing energy.

2. DIBLOCK COMPATIBILIZERS

Diblock copolymers represent the simplest possible compatibilizer. As such, they are a valuable case study. First, we consider the scaling of toughness as a function of the areal density of compatibilizers (Σ) for long diblock compatibilizers at $\chi N = 650$ with a reference volume of the size of the monomer ($= 1/\rho_A = 1/\rho_B$, where ρ_m is the number density of species m) in Fig. S3 to determine if it is consistent with Brown's theory which predicts toughness scales as Σ^2 (28). The solid guiding line demonstrates that our data aligns with this scaling. Next, we consider the SCFT parameterized theory. This model goes to zero for the smallest Σ but aligns closely with Brown's predictions and the data in the other cases.

We now turn to whether one needs to consider load-bearing entanglements rather than all entanglements. Here, we consider short diblock compatibilizers with a block length of $L = 80$ monomers and homopolymers of length $N = 256$ monomers. We fix $N_e = 10.3$ monomers, set $\chi N = 650$, and vary the areal density of these compatibilizers from $\Sigma = 0.036/\sigma^2$ to $\Sigma = 0.18/\sigma^2$. As in Fig. 3 in the Main Text, each point is the average of 3 replicates, and error bars denote the standard error. These shorter diblocks are sensitive to whether the first and last primitive paths are removed,

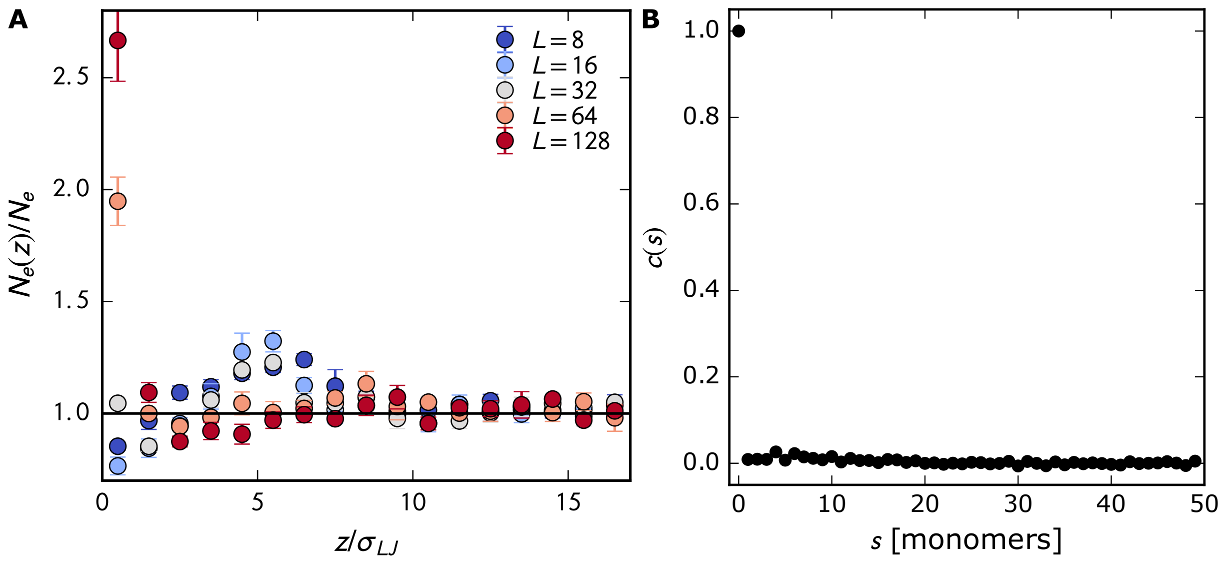

Fig. S1. SCFT parameterization assumptions. (**A**) This figure plots the change in the entanglement length scale as a function of the distance away from the interface ($N_e(z)$) normalized by the bulk N_e at $\Sigma = 0.045\sigma_{LJ}^{-2}$ and $\chi N = 650$. The block lengths L are listed in monomers. These results depend on Σ, χ, and compatibilizer architecture. Error bars represent standard errors of 3 replicates. (**B**) This image shows entanglement correlations calculated from Eq. S1 for compatibilizers with a block length of $L = 32$ monomers at $\Sigma = 0.045\sigma_{LJ}^{-2}$ and $\chi N = 650$.

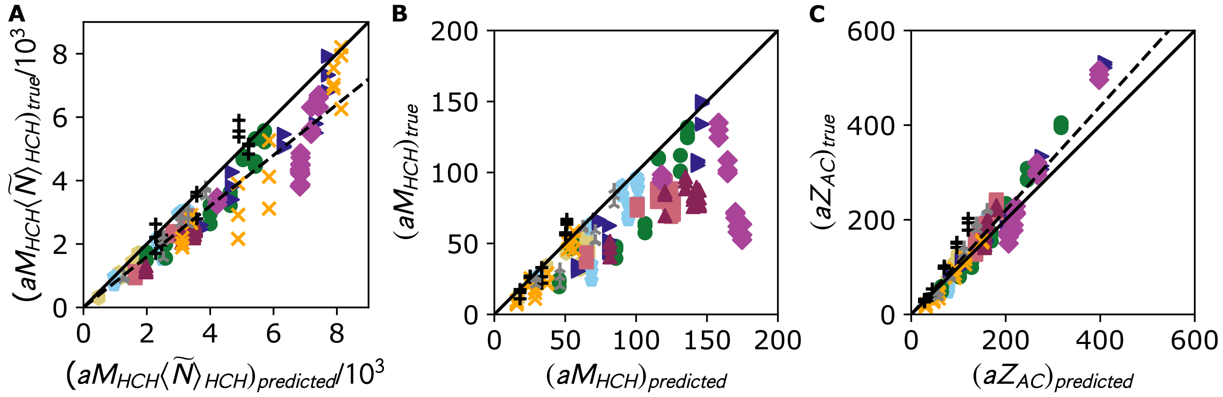

Fig. S2. Microscopic variables true against predicted. Plots show the SCFT predictions against measured molecular dynamics values of (**A**) $M_{HCH}\langle\widetilde{N}\rangle_{HCH}$, (**B**) M_{HCH}, and (**C**) Z_{AC}. These values are normalized by the interfacial area (a). Points correspond to individual simulations. Symbol colors and shapes correspond to Fig. 3 in the Main Text. Dashed lines in (**A**) and (**C**) represent 20% and 10% deviations from true values, respectively.

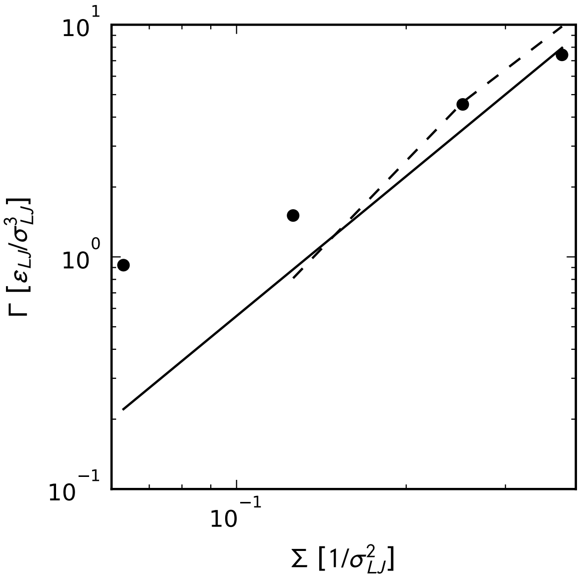

Fig. S3. Long diblock toughness scaling. Image shows mean simulation points and the model presented in the Main Text as a dashed line. The black line denotes a $\Gamma \propto \Sigma^2$ power law, consistent with Brown's theory and measurements.

making them an excellent test of whether to keep the first and last primitive paths. When we use the original model with load-bearing entanglements, the true toughness values are well-predicted by the model that works with the significantly longer compatibilizers, as shown in Fig. S4. Because these compatibilizers are not so affected by removing their first and last primitive paths, this matching indicates that using load-bearing entanglements in our model is reasonable. On the other hand, when we apply the model with all entanglements, toughness is significantly overpredicted in all cases. Thus, using load-bearing entanglements allows our theory to work for considerably smaller compatibilizers.

3. TOUGHNESS OF POLYMERS OF THE SAME LENGTH

While Fig. 4C increases N such that N/N_e is roughly constant, one may ask how toughness changes when N is kept constant. This question is not straightforward. In Fig. S5, we perform simulations in which we vary N_e by varying chain stiffness but keep polymer length fixed at $N = 256$ monomers for two different block lengths $L = 128$ and $L = 32$ monomers. Figure S5A shows that if the block-copolymer blocks are long enough ($L \gg N_e$), blend toughness increases with N_e because this will lengthen the load-bearing strands. However, Fig. S5B illustrates if the blocks are short, changing N_e can cause a decrease in the number of load-bearing strands. This effect occurs because the blocks are not long enough to entangle with the homopolymer matrix, causing blend toughness to decrease.

4. MONTE CARLO PROCEDURE FOR INITIAL POLYMER CONFIGURATIONS

For our initial homopolymer configurations, we fix bond length to r_b, the minimum of the FENE potential. Starting with a single monomer, we grow polymers of length N by adding monomers sequentially such that they have a bond length r_b and a polar bond angle (θ) chosen from the distribution

$$P(\theta) = \frac{\sin(\theta)\, e^{-U_\theta(\theta)/k_B T}}{\int_0^\pi d\theta \sin(\theta)\, e^{-U_\theta(\theta)/k_B T}}, \tag{S3}$$

where

$$U_\theta(\theta) = \kappa(1 - \cos(\theta)) + \epsilon_{LJ}\left(\left(\frac{\sigma_{LJ}}{l(\theta)}\right)^{12} - \left(\frac{\sigma_{LJ}}{l(\theta)}\right)^{6}\right), \tag{S4}$$

$T = \epsilon_{LJ}/k_B$ is our equilibration temperature, and $l(\theta) = r_b\sqrt{2 + 2\cos(\theta)}$. Here, ϵ_{LJ} and σ_{LJ} are the well-depth and length scale of the Lennard-Jones potential in our molecular dynamics simulations. The first term of Eq. S4 controls the polymer's stiffness, while the second term of Eq. S4 approximates the non-bonded interactions of the next-nearest neighbor, making our random walk non-reversible. Given a fixed r_b, the distance between a monomer and its next-nearest neighbor is $l(\theta)$. We fix the stiffness of these polymers, κ, to match the end-to-end distances found in Auhl *et al.* (55).

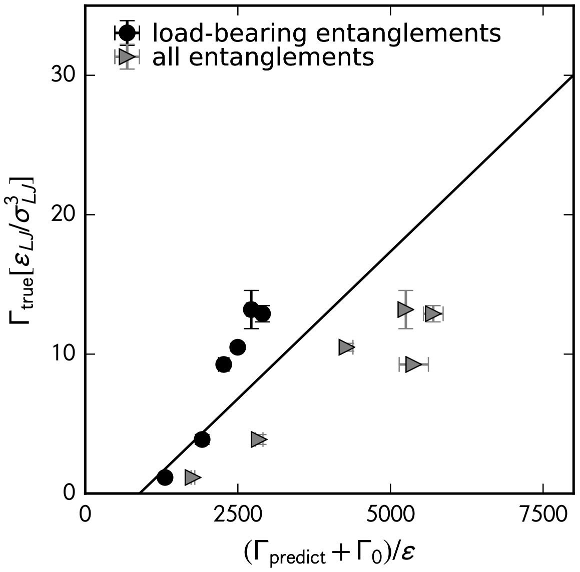

Fig. S4. Validation of the use of load-bearing entanglements. This plot shows the true toughness against predicted toughness for a set of polymer blends compatibilized by short diblocks in which Σ is varied. Black circles show the predictions when using load-bearing entanglements in the model. Grey triangles show the same results when reanalyzed with all entanglements. The black line is the fit shown in Fig. 3.

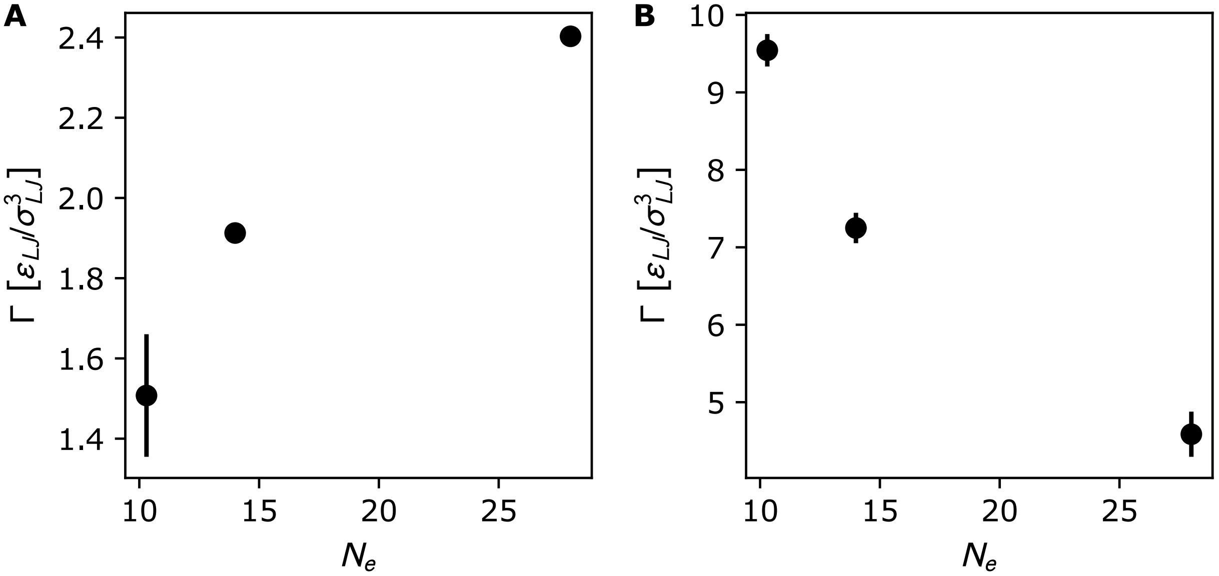

Fig. S5. Toughness of polymers of same length This plot illustrates toughness (Γ) against entanglement length (N_e) for polymers of length $N = 256$ and $\epsilon_{AB} = 0.5$ monomers and block lengths of (**A**) $L = 128$ and (**B**) $L = 32$ monomers. Error bars are standard errors across three replicates.

We begin the initialization of our compatibilizers using the same method as the homopolymers, treating all monomers as the same type. We then place their center of mass on the interface ($z = 0$). To equilibrate them fully, we perform Monte Carlo moves on our old compatibilizer configurations to generate new configurations. The Monte Carlo moves we choose are (i) solid rotations, (ii) azimuthal rotations of bond angles (ϕ), and (iii) polar rotations of bond angles (θ). The solid rotations of the compatibilizer from the initial configuration are chosen from a Gaussian distribution with a standard deviation of 0.05 radians. Azimuthal rotations are chosen from a Gaussian distribution with a standard deviation of 0.35 radians from the initial angle. Angles for the polar rotations of bond angles are redrawn from Eq. S3. After each of these moves, the center of mass is shifted back to the interface.

Assuming a homogeneous field of homopolymer A for $z < 0$ and homopolymer B for $z > 0$, it can be shown that the energy of an A monomer a distance z from the interface is

$$U_A(z)/U_0 = \frac{-2}{9}\left(1 + \epsilon_{LJ}^{AB}/\epsilon_{LJ}\right) + \left(1 - \epsilon_{LJ}^{AB}/\epsilon_{LJ}\right)\begin{Bmatrix} \frac{3z}{20\sigma_{LJ}}, & \text{if } |\frac{z}{\sigma_{LJ}}| \leq 1 \\ \text{sign}(z)\left(\frac{2}{9} - \frac{1}{12}|\frac{\sigma_{LJ}}{z}|^3 + \frac{1}{90}|\frac{\sigma_{LJ}}{z}|^9\right), & \text{if } |\frac{z}{\sigma_{LJ}}| > 1 \end{Bmatrix}, \tag{S5}$$

where $U_0 = 2\pi\epsilon_{LJ}\rho\sigma_{LJ}^3$ is an overall energy scale, ϵ_{LJ}^{AB} is the Lennard-Jones well-depth of the A-B species, and $\rho = \rho_A = \rho_B$ is the system density of species A and B. We perform this derivation by integrating the non-bonded Lennard-Jones interaction over all space except a sphere of excluded volume around the monomer with a σ_{LJ} radius. A similar expression can be derived for a B monomer, $U_B(z)$, where the second term has the opposite sign. Thus, the non-bonded energy compatibilizer energy is

$$U_{n.b.} = \sum_{i=1}^{N} U_{m_i}(z_i), \tag{S6}$$

where m_i is the type of monomer i and z_i is the distance of monomer i away from the interface. The Monte Carlo acceptance criteria is then

$$P_{accept} = \min\left(1, \exp\left(-\frac{U_{n.b.,new} - U_{n.b.,old}}{k_B T}\right)\right) \tag{S7}$$

We repeat these Monte Carlo moves 10^6 times for each compatibilizer, at which point $U_{n.b.}$ plateaus.

We randomly place these compatibilizers in the xy plane at the interface. As a final step in generating our initial configurations, we perform 300 small solid rotations and translations to each homopolymer to minimize density fluctuations in the box as suggested in Auhl *et al.* (55).

5. FLORY-HUGGINS PARAMETERS OF MOLECULAR DYNAMICS SIMULATIONS

We run additional simulations of our polymer blends without compatibilizer to obtain our molecular dynamics simulations' Flory-Huggins parameters. We measure the interfacial width between the A and B species as shown in Fig. S6. We then determine χ by fitting these interfacial profiles using analytic expressions provided by Semenov (53):

$$\phi_A(z) = \frac{1}{2}\left(1 + \tanh(2z/\Delta)\right), \tag{S8}$$

where ϕ_A is the volume fraction of species A, z is the distance from the interface, and

$$\Delta = \sqrt{\frac{2R_{ee}^2}{3\chi N - 12\ln(2)}}. \tag{S9}$$

Here, R_{ee} is the end-to-end distance of the neat homopolymer. This expression assumes two homopolymers of the same length N. We take the value of $R_{ee} = 29.1\sigma_{LJ}$ from neat homopolymer simulations with a bond angle rigidity $\kappa = 2\epsilon$. This value is commensurate with the $\kappa = 2\epsilon$ simulations of Auhl *et al.* (55). We find excellent agreement between the analytical theory and our simulations.

6. THE EFFECT OF VARYING NON-BONDED CUTOFF RADIUS ON TOUGHNESS

In addition to the effect of entanglements on toughness, one can ask what effect our attractive component plays in transferring stress through entanglements and across load-bearing strands. To investigate, we take equilibrated, glassy configurations for a select set of simulations and modify their Lennard-Jones cutoff radius from $R_c = 2.5\sigma_{LJ}$ to $R_c = 1.5\sigma_{LJ}$. We then strain these simulations to failure using the same protocol as described previously and compare their toughness to that of the $R_c = 2.5\sigma_{LJ}$ simulations in Fig. S7. These simulations do not suggest a systematic deviation from a perfectly correlated $\Gamma_{R_c=2.5\sigma_{LJ}} = \Gamma_{R_c=1.5\sigma_{LJ}}$ relationship. These results suggest the attractive component in our simulations has little bearing on stress transfer, indicating topological constraints are much more important.

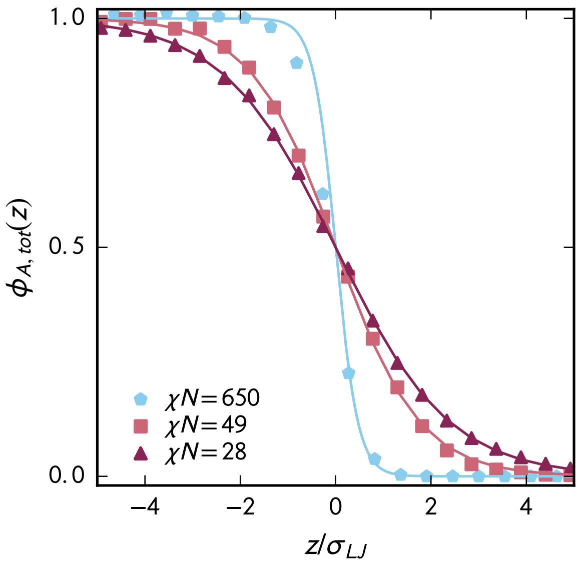

Fig. S6. Interfacial profiles. Plots of the interfacial profiles of a set of simulations without compatibilizers in which $\epsilon_{LJ}^{AB} = 0.5$ (blue pentagons), 0.97 (pink squares), and $0.985\epsilon_{LJ}$ (red triangles). Points correspond to symbols used in Fig. 3 in the Main Text. The lines correspond to fits to analytic expressions by Semenov (53).

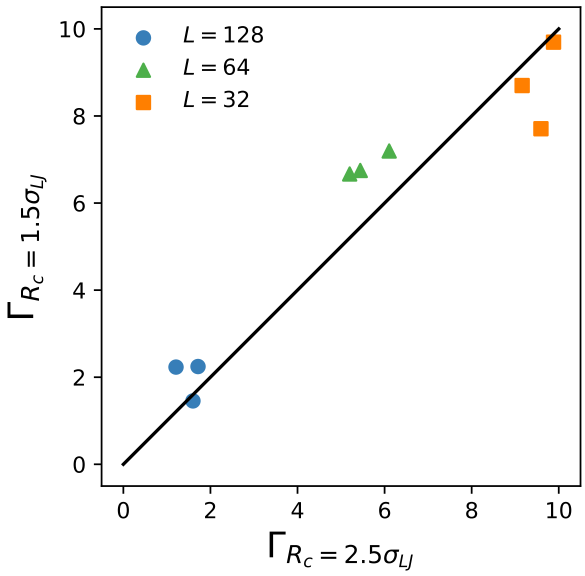

Fig. S7. Toughness comparison for $R_c = 2.5\sigma_{LJ}$ and $R_c = 1.5\sigma_{LJ}$. Image plots toughnesses for simulations for block lengths $L = 32$, $L = 64$, and $L = 128$ monomers for our ($N = 256$, $\chi N = 650$) polymers. Before deformation, we modify the cutoff of the nonbonded Lennard-Jones potential from $R_c = 2.5\sigma_{LJ}$ to $R_c = 1.5\sigma_{LJ}$ and compare their toughnesses, $\Gamma_{R_c=2.5\sigma_{LJ}}$ and $\Gamma_{R_c=1.5\sigma_{LJ}}$. The line represents a perfect $\Gamma_{R_c=2.5\sigma_{LJ}} = \Gamma_{R_c=1.5\sigma_{LJ}}$ relationship. We see no systematic deviation from this relationship.

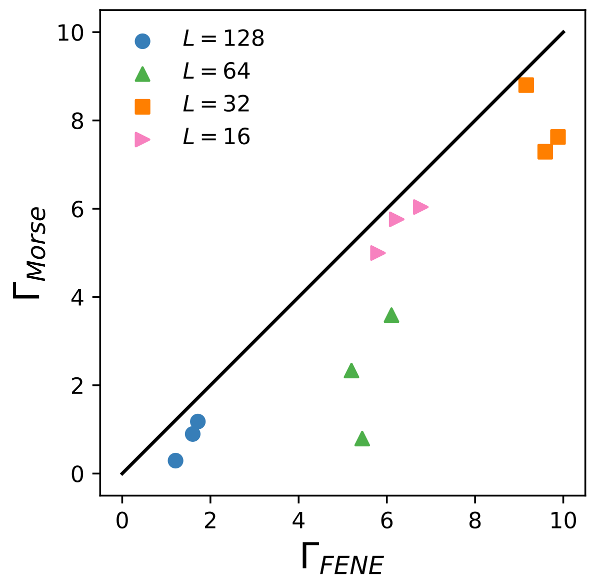

Fig. S8. Toughness comparison for FENE and Morse bonds. Image plots toughnesses for simulations with block lengths $L = 16$, $L = 32$, $L = 64$, and $L = 128$ monomers for our ($N = 256$, $\chi N = 650$) polymers. Before deformation, we modify the bonded potential from a FENE to a Morse potential. The line represents a perfect $\Gamma_{FENE} = \Gamma_{Morse}$ relationship.

7. THE EFFECT OF THE BONDED POTENTIAL ON TOUGHNESS

The Morse potential is a convenient bonding potential that better approximates the potential energy of a diatomic molecule than harmonic bonds. As such, one can ask how changing from a FENE to a Morse-like potential would change the results of our work. Analogous to the previous section, we take glassy configurations for a select set of simulations and modify their bonding potential to a Morse potential before strain. Specifically, we used $U_b(r) = D\left(1 - e^{-\alpha(r-r_0)}\right)^2$, where $D = 17.3\epsilon$, $\alpha = 10.0/\sigma_{LJ}$, and $r_0 = 0.961\sigma_{LJ}$. This potential has the same minimum as the FENE potential and the same energy difference at $r = 1.2\sigma_{LJ}$, at which point the bond permanently breaks. We strain these simulations to failure and compare their toughness to that of the FENE bonded strain simulations in Fig. S8. Here, we find a small drop in the toughness of the compatibilized blends. Still, overall, the toughness of simulations with this bonded potential strongly correlates with the FENE-bonded simulations. These results suggest that switching to a Morse-like potential may quantitatively change parameters such as ϵ but will not modify our theory.
